# Antibiotic Resistance Patterns of Bacteria Involved in Colonization and/or Infection of Patients in Intensive Care Units in Northeastern Romania

**DOI:** 10.3390/antibiotics14111063

**Published:** 2025-10-23

**Authors:** Alexandru Duhaniuc, Cristina Mihaela Sima, Georgiana Buruiană, Cătălina Luncă, Olivia Simona Dorneanu

**Affiliations:** 1 Department of Preventive Medicine and Interdisciplinarity—Microbiology, Grigore T. Popa University of Medicine and Pharmacy, 700115 Iasi, Romania; alexandru.duhaniuc@umfiasi.ro (A.D.); cristina.sima@umfiasi.ro (C.M.S.); georgiana.buruiana@umfiasi.ro (G.B.); catalina.lunca@umfiasi.ro (C.L.); 2 Regional Center of Public Health, National Institute of Public Health, 700465 Iasi, Romania; 3 Clinical Hospital of Infectious Diseases “Sf. Parascheva”, 700116 Iasi, Romania; 4 Emergency Clinical Hospital for Children “Sf. Maria”, 700309 Iasi, Romania

**Keywords:** host colonization, infection, MDR bacteria, ICU, antimicrobial resistance

## Abstract

Background/Objectives: Healthcare-associated infections caused by multidrug-resistant (MDR) bacteria pose a significant and growing public health challenge, particularly in high-risk settings such as Intensive Care Units (ICUs). Colonization is often asymptomatic but can precede infection and contribute to hospital outbreaks, making early detection critical for infection control and containment. The aim of the study is to evaluate the antibiotic susceptibility patterns of MDR bacteria involved in colonization and/or infection among patients admitted to the ICU at a tertiary care hospital in Northeastern Romania and to investigate the relationship between MDR bacterial colonization and subsequent infection. Methods: A total of 118 patients from ICU were included in this study and a total of 609 bacterial strains were isolated, involved in both colonization and infection, with multiple isolates per patient included if obtained from different sites or time points. Results: A predominance of Gram-negative bacilli was found to be involved in both colonization and infection in the ICU, with *Acinetobacter baumannii* (*n* = 146; 26.64%), *Klebsiella pneumoniae* (*n* = 146; 26.64%), *Escherichia coli* (*n* = 60; 10.95%), and *Pseudomonas aeruginosa* (*n* = 56; 10.22%) as the main bacteria involved in colonization, and *A. baumannii* (*n* = 23; 37.7%), *K. pneumoniae* (*n* = 19; 31.1%), and *P. aeruginosa* (*n* = 15; 24.6%) as the main bacteria involved in infections. The study revealed a high diversity of antibiotypes among *K. pneumoniae* (43 distinct antibiotypes), *E. coli* (35 distinct antibiotypes) and *P. aeruginosa* (27 distinct antibiotypes). In contrast, only 6 antibiotypes were identified for *A. baumannii*, with most strains belonging to a single dominant antibiotype. For *K. pneumoniae*, *E. coli* and *A. baumannii*, infections mainly involved the same antibiotype as that found in colonization, while infections with *P. aeruginosa* were often linked to different antibiotypes than those involved in colonization. Conclusions: This study demonstrates a high colonization-to-infection link among ICU patients in a Northeastern Romanian tertiary care hospital, and these findings underscore the importance of systematic colonization screening to identify patients at high risk in ICU settings.

## 1. Introduction

Healthcare-associated infections caused by multidrug-resistant (MDR) bacteria pose a significant and growing public health challenge, particularly in high-risk settings such as Intensive Care Units (ICUs), and especially in ICUs from infectious disease hospitals where patients frequently receive broad-spectrum antibiotic therapy [1,2]. These infections are notoriously difficult to treat due to limited antimicrobial options and are associated with increased morbidity, mortality, and healthcare costs [3,4,5]. MDR bacteria are typically defined as bacteria resistant to at least three classes of antimicrobial agents, with key pathogens including *Staphylococcus aureus*, *Enterococcus* spp., *Enterobacterales*, *Pseudomonas aeruginosa*, and *Acinetobacter* spp. [6]. ICU patients are especially vulnerable due to prolonged hospital stays, frequent invasive procedures, and immunosuppression, all of which facilitate colonization and infection by MDR bacteria [7,8,9]. Colonization is often asymptomatic but can precede infection and contribute to hospital outbreaks, making early detection critical for infection control and containment [10,11].

Antimicrobial resistance (AMR) is currently recognized as one of the most significant threats to global health, necessitating immediate and coordinated action to curb its progression. The World Health Organization (WHO) has identified antimicrobial resistance as a critical public health concern and has developed a priority list of resistant pathogens based on factors such as infection-associated mortality, transmissibility, availability of effective treatments, and the feasibility of prevention in both healthcare and community settings. This prioritization aims to guide research and stimulate the development of new antimicrobial agents targeting the most urgent threats. The WHO list categorizes MDR bacteria into three priority tiers: critical, high, and medium. The critical-priority group includes carbapenem-resistant *Enterobacterales*, third-generation cephalosporin-resistant *Enterobacterales*, and carbapenem-resistant *Acinetobacter baumannii*. The high-priority category encompasses vancomycin-resistant *Enterococcus faecium*, carbapenem-resistant *Pseudomonas aeruginosa*, methicillin-resistant *Staphylococcus aureus* (MRSA), fluoroquinolone-resistant *Salmonella Typhi*, non-typhoidal *Salmonella*, and *Shigella* species. The medium-priority group includes macrolide-resistant *Streptococcus pneumoniae* and ampicillin-resistant *Haemophilus influenzae* [12].

AMR continues to pose a critical challenge to public health across Europe, with MDR bacteria contributing significantly to the burden of healthcare-associated infections (HAIs). An estimated 80,000 hospitalized patients suffer from at least one HAI on any given day, contributing to approximately 16 million additional hospital days annually. The prevalence of HAIs in high-income countries is around 7.5%, while in low- and middle-income countries it ranges between 5.7% and 19.2%. In Romania, HAIs remain a significantly underrecognized problem, with official reports indicating prevalence rates of only 0.2–0.25%, largely due to substantial underreporting. Across the EU, it is estimated that more than 4 million patients acquire a nosocomial infection each year, resulting in approximately 37,000 deaths [13,14].

Surveillance data from the European Centre for Disease Prevention and Control (ECDC) highlight alarming trends in the prevalence of resistance among key bacterial pathogens. Between 2016 and 2020, there was a marked increase in carbapenem-resistant *E. coli* and *Klebsiella pneumoniae*, as well as vancomycin-resistant *E. faecium* across the region [15]. By 2021, over half of *E. coli* strains and approximately one-third of *K. pneumoniae* strains exhibited resistance to at least one class of antibiotics, with combined resistance to multiple classes being increasingly common [16]. The prevalence of carbapenem resistance in *K. pneumoniae* has been particularly concerning. In 2021, over 25% of European countries reported carbapenem resistance rates exceeding 10%, and by 2022, these rates had increased by nearly 50% compared to the previous year. *E. coli* and *K. pneumoniae* remained the most frequently reported bacterial species causing bloodstream infections (BSIs) in Europe, with *E. coli* accounting for 39.2% and *K. pneumoniae* for 12.3% of cases in 2022 [16,17]. Although some declines in *E. coli* resistance to third-generation cephalosporins and in methicillin-resistant *S. aureus* (MRSA) BSIs were noted from 2019 to 2022, the incidence of carbapenem-resistant *K. pneumoniae* continued to rise, signaling an urgent need for targeted interventions [15,16,17]. In 2023, *E. coli* and *K. pneumoniae* remained the top contributors to bloodstream infections involving antimicrobial resistance in the European Union [18]. While the overall resistance incidence in *E. coli* declined compared to 2019, levels have been rising since 2021 and may soon return to previous highs. Conversely, AMR incidence in *K. pneumoniae* has already surpassed 2019 levels, showing a consistent upward trend from 2019 to 2023. Notably, carbapenem resistance in *K. pneumoniae* remains a primary concern, with higher average resistance rates than those observed for *E. coli*, despite the latter’s higher infection incidence. Other Gram-negative pathogens, including *P. aeruginosa* and *Acinetobacter* spp., exhibited lower overall incidence rates but demonstrated worrisome resistance trends, with a significant upward trend in resistance to carbapenems, piperacillin-tazobactam, and ceftazidime in *P. aeruginosa*. While some antimicrobial resistance trends appear to be stabilizing or even declining, particularly in Northern and Western Europe, countries in Southern and Eastern Europe consistently report higher rates of AMR and associated bloodstream infections. These geographic disparities underscore the need for regionally tailored surveillance and infection control strategies to effectively contain the spread of MDR bacteria across the continent [15,16,17,18]. In Romania, AMR is a major public health challenge, mainly due to excessive and often inappropriate use of antimicrobials. Romania ranks as the second-highest country among EU/EEA countries in total consumption (community and hospital sectors combined) of antibiotics for systemic use with 27.4 DDD per 1000 inhabitants per day. This led to high resistance rates in clinically relevant pathogens, such as *S. aureus*, *K. pneumoniae*, *P. aeruginosa*, and *A. baumannii* [18]. According to the most recent EU/EEA annual report on antimicrobial resistance, Romania has reported concerning trends between 2019 and 2023. An increasing trend in carbapenem resistance was observed for *E. coli* and *K. pneumoniae*; however, the resistance rates remained low for *E. coli* (<2%), while for *K. pneumoniae*, resistance rates for carbapenems reached 52.8%. *K. pneumoniae* also shows alarmingly high resistance rates for third-generation cephalosporins (69.4%), fluoroquinolones (64%) and aminoglycosides (57.1%). In *Acinetobacter* spp., resistance rates exceed 80% for carbapenems, fluoroquinolones, and aminoglycosides, although a declining trend has been observed for fluoroquinolones. Similarly, MRSA has shown a downward trend but remains prevalent at 39.4%. *P. aeruginosa* continues to display high resistance rates to piperacillin & tazobactam (50%), ceftazidime (48.6%), carbapenems (52.7%), fluoroquinolones (49.2%), and aminoglycosides (44.2%). Moreover, combined resistance to ≥3 antimicrobial groups remain widespread, particularly in *K. pneumoniae* (54.4%), *P. aeruginosa* (49.5%), and *Acinetobacter* spp. (81.6%) [17,18]. These data underscore the significant burden of MDR bacteria in Romania and raise concerns about therapeutic limitations and infection control strategies.

Despite these worrying trends, important surveillance gaps remain. National AMR data are frequently incomplete, with insufficient details regarding regional variation, hospital context, or specific patient groups. Routine surveillance in Romania tends to underrepresent colonization data, and information linking colonization with subsequent infection is especially scarce. This gap in knowledge hampers the implementation of effective infection prevention and control measures and limits the ability of stewardship programs to adapt to local resistance patterns. Given the country’s high antibiotic consumption and high rates of resistance in critical pathogens, local ICU-focused surveillance is essential. Generating such data not only informs patient management at the institutional level but also contributes to the broader understanding of regional resistance dynamics. The aim of the study is therefore to evaluate the antibiotic susceptibility patterns of MDR bacteria involved in colonization and/or infection among patients admitted to the ICU at a tertiary care hospital in Northeastern Romania, and to investigate if there is a relationship between MDR bacterial colonization and subsequent infection. By addressing local surveillance gaps, this work provides much-needed insights into the burden of MDR bacteria in Romanian ICUs and strengthens the evidence base for antimicrobial stewardship and infection prevention strategies in high-risk clinical settings.

## 2. Results

### 2.1. Distribution of Bacterial Strains

A total of 118 patients from ICU were included in this study and a total of 609 bacterial strains were isolated, involved in both colonization (548 strains) and infection (61 strains). All 118 patients were screened for MDR bacteria upon ICU admission and 116 patients were colonized with at least 1 MDR bacteria, while two patients had no colonization with MDR bacteria; however, they acquired MDR bacteria during their ICU stay. Out of the total of 118 patients, only 42 patients were followed-up to assess changes in colonization, with the number decreasing at each subsequent screening point (Table 1). All patients acquired at least one additional MDR bacteria while staying in the ICU.

Thirty-three patients (27.97%) developed infections while staying in the ICU. From the total number of bacterial strains isolated, 548 strains (89.98%) were involved in colonization and 61 strains (10.02%) were isolated from infections. Regarding bacteria involved in colonization, 465 (84.85%) strains were Gram-negative bacteria, while 83 (15.15%) were Gram-positive bacteria. The majority of bacteria (*n* = 59; 96.72%) isolated from infections were represented by Gram-negative bacteria, and only 2 strains (3.28%) were Gram-positive bacteria (Table 2).

From the total number of strains involved in colonization, 193 (35.22%) were isolated from pharyngeal exudate, 93 (16.97%) from nasal exudate, and 262 (47.81%) from rectal swabs. From the bacterial strains involved in infections, 31 (50.82%) were isolated from tracheal aspirates, 15 (24.59%) from urine, 13 (21.31%) from blood, 1 (1.64%) from sputum and 1 (1.64%) from conjunctival exudate (Table 3).

### 2.2. Colonization Patterns with Bacteria in ICU Patients

**Figure 1:**
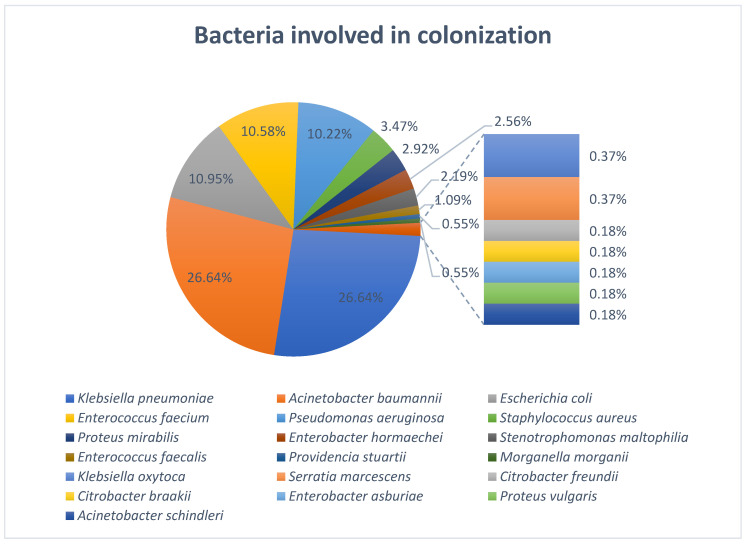
Overview of bacterial species involved in colonization.

The most frequent bacteria responsible for colonization in the ICU were *Acinetobacter baumannii* (*n* = 146; 26.64%) and *Klebsiella pneumoniae* (*n* = 146; 26.64%), followed by *Escherichia coli* (*n* = 60; 10.95%), *Enterococcus faecium* (*n* = 58; 10.58%), *Pseudomonas aeruginosa* (*n* = 56; 10.22%), and *Staphylococcus aureus* (*n* = 19; 3.47%) (Figure 1).

Upon admission to the ICU, *K. pneumoniae* (*n* = 65) and *A. baumannii* (*n* = 56) were the most frequently isolated bacteria, followed up by *E. coli* (*n* = 49) and *E. faecium* (*n* = 42). A variety of other MDR bacteria were also found being involved in colonization upon ICU admission; however, these were less frequently isolated. The main organism isolated from pharyngeal and nasal swabs was *A. baumannii*, while *Enterobacterales*, such as *K. pneumoniae* and *E. coli*, and also *E. faecium* were the main organisms isolated from rectal swabs (Table S1). At 7 days after ICU admission the most prevalent bacteria involved in colonization were *A. baumannii* (*n* = 55) and *K. pneumoniae* (*n* = 48), making up to two thirds (67.76%) of colonization cases (Table S2). After 14 days of ICU stay, the main colonizers remained *A. baumannii*, *K. pneumoniae* and *P. aeruginosa* (Tables S3–S6).

**Figure 2:**
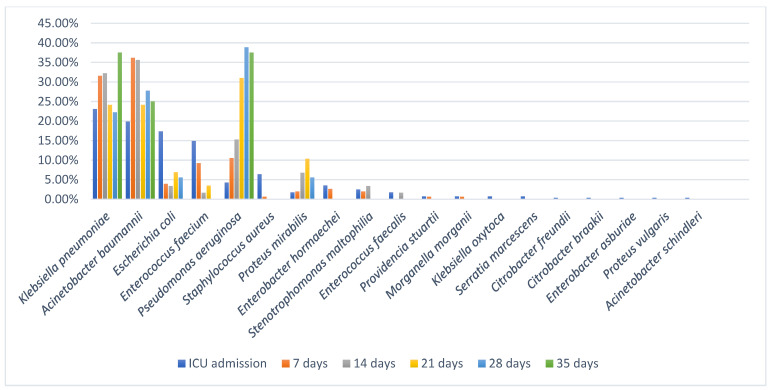
Dynamics of bacteria involved in colonization through different time points. The number of patients screened at each time point declined over time: all 118 patients were screened at ICU admission, 42 patients at 7 days, 14 patients at 14 days, 8 patients at 21 days, 2 patients at 28 days, and 1 patient at 35 days. To account for the decreasing number of patients over time, the data on the *y*-axis have been normalized by the number of patients screened at each respective time point.

At ICU admission, a total of 299 colonizing strains (54.56%) were detected among the 118 patients, and during the ICU stay, 249 additional strains (45.44%) were identified among smaller cohorts at subsequent time points: 151 strains among 27 patients at 7 days, 63 strains among 8 patients at 14 days, 29 strains among 4 patients at 21 days, 18 strains among 2 patients at 28 days and 8 strains in 1 patient at 35 days after ICU admission. The most frequently isolated species were *K. pneumoniae* and *A. baumannii*, each accounting for 146 strains (26.64%). While both species were present at ICU admission (65 and 56 strains, respectively), a larger proportion was acquired during ICU stay: 81 (55.48%) strains post-admission for *K. pneumoniae* (χ^2^ = 1.75, *p* = 0.186) and 90 (61.64%) strains post-admission for *A. baumannii* (χ^2^ = 7.92, *p* ≈ 0.0049). *P. aeruginosa* (*n* = 56; 10.22%) also exhibited a strong ICU-associated pattern (χ^2^ = 18.29, *p* = 0.000019), with 44 strains (78.57%) acquired during ICU stay. In contrast, *E. coli* (*n* = 60; 10.95%) was predominantly present at ICU admission (*n* = 49; 81.67%), with a low post-admission acquisition rate (*n* = 11; 18.33%) (χ^2^ = 24.07, *p* ≈ 0.00000093). Although fewer patients were available for follow-up at later time points, those who remained in the ICU beyond 14–21 days demonstrated markedly higher colonization density, particularly with *A. baumannii*, *K. pneumoniae*, and *P. aeruginosa*. For instance, by day 28, the two patients assessed carried a combined total of 18 colonizing strains, reflecting cumulative microbial burden under prolonged ICU exposure. *E. faecium* (n = 58; 10.58%) was detected in 42 patients at admission, and its post-admission acquisition rate was lower than that of major Gram-negative organisms. Other organisms were detected at lower frequencies, including *S. aureus* (*n* = 19; 3.47%), *P. mirabilis* (*n* = 16; 2.92%), *E. hormaechei* (*n* = 14; 2.56%), *S. maltophilia* (*n* = 12; 2.19%) and *E. faecalis* (*n* = 6; 1.09%). Rarely isolated species (≤3 strains each) included *K. oxytoca*, *P. stuartii*, *M. morganii*, *Citrobacter* spp., *S. marcescens*, *P. vulgaris*, and *A. schindleri*, each contributing <1% of total isolates (Figure 2).

### 2.3. Infections with MDR Bacteria in ICU Patients

**Figure 3:**
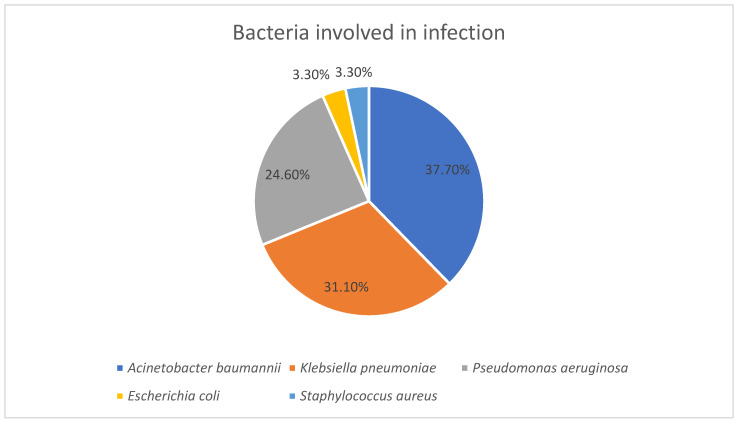
Bacterial species involved in infections.

Thirty-three patients developed an infection while staying in the ICU. Regarding bacteria isolated from infections, the most frequently isolated was *A. baumannii* (*n* = 23; 37.7%), followed by *K. pneumoniae* (*n* = 19; 31.1%) and *P. aeruginosa* (*n* = 15; 24.6%). Less frequently isolated strains were represented by *E. coli* (*n* = 2; 3.3%) and *S. aureus* (*n* = 2; 3.3%) (Figure 3).

The majority of strains (*n* = 32; 52.5%) were involved in lower respiratory tract infections with *A. baumannii* accounted for most cases (*n* = 17; 53.1%). The rest of the strains were involved in catheter-associated urinary tract infections (*n* = 15; 24.6%) and bloodstream infections (*n* = 13; 21.3%). Frequently isolated from urinary tract infections and blood cultures was *K. pneumoniae* (*n* = 10; 66.7% and *n* = 5; 38.5% respectively) (Table 4).

Notably, 29 out of the 33 infected patients (88%) had prior colonization with the same bacterial species responsible for their infection. A total of 4 out of 29 patients had an infection with the same bacterial species involved in colonization but with a different antibiotype, while most patients (*n* = 25) had at least an infection with a strain presenting the same antibiotype with the colonizing strain. Infections caused by *K. pneumoniae*, *A. baumannii*, and *P. aeruginosa* were most frequently associated with prior colonization, particularly at multiple anatomical sites such as the pharynx, intestine, and nasal cavity. While most infections with *K. pneumoniae* and *A. baumannii* involved the same antibiotype (10 and 22 strains, respectively), infections with *P. aeruginosa* mostly involved different antibiotypes (9 strains) than the colonizing strains. In contrast, a minority of infections (12%) occurred in patients who had no documented colonization with the infecting organism (Table 5).

Among patients with respiratory tract infections (*n* = 24), 20 (83.3%) were colonized with bacteria on the pharyngeal or nasal mucosa, while 4 (16.7%) had no such colonization. A chi-square test demonstrated a statistically significant association between respiratory tract infections and pharyngeal/nasal colonization (χ^2^ = 4.54, *p* = 0.033), suggesting that colonization at these sites may be a risk factor for subsequent respiratory tract infections. In contrast, among patients with urinary tract infections (UTIs) (*n* = 19), 12 (63.2%) had intestinal colonization, whereas 7 (36.8%) did not. Statistical analysis using the chi-square test revealed no significant association between intestinal colonization and UTIs (χ^2^ = 0.001, *p* = 0.974).

### 2.4. Antibiotic Resistance Patterns in Enterobacterales

#### 2.4.1. Antibiotic Resistance Patterns of *K. pneumoniae* Involved in Colonization and Infection

All strains of *K. pneumoniae* showed a high level of multidrug resistance. *K. pneumoniae* strains exhibited near-universal resistance to most β-lactams, fluoroquinolones, aminoglycosides (except amikacin), and trimethoprim & sulfamethoxazole. Carbapenem resistance was high, particularly to ertapenem and imipenem, but lower for meropenem. Ceftazidime & avibactam showed moderate activity, while colistin remained the most effective agent, with the majority of strains being susceptible. (Table 6).

The antimicrobial susceptibility testing of *K. pneumoniae* strains isolated from infections revealed widespread resistance across multiple antibiotic classes. *K. pneumoniae* isolates showed universal resistance to most β-lactams, fluoroquinolones, and trimethoprim & sulfamethoxazole, with high resistance also to piperacillin & tazobactam and aztreonam. Resistance to carbapenems varied: imipenem (73.7%), imipenem & relebactam (68.4%), and meropenem (63.1%), indicating activity in a subset of isolates. Ceftazidime & avibactam and amikacin retained partial activity, while colistin remained the most effective agent, with the majority of strains being susceptible (68.4%) (Table 7). For *K. pneumoniae*, the Multiple Antibiotic Resistance (MAR) index values ranged from 0.34 to 1, indicating a high level of resistance across the strains. Only a small proportion of isolates exhibited lower MAR indices (<0.5), including 1 strain at 0.34, 2 strains at 0.39, 3 strains at 0.43, and 2 strains at 0.47. The majority of isolates displayed high MAR indices (>0.7). The most frequent values were 0.73 (*n* = 30), 0.82 (*n* = 31), 0.86 (*n* = 14), 0.95 (*n* = 39), and 1 (*n* = 19).

All 165 strains of *K. pneumoniae* were classified into 43 antibiotypes. While the majority of antibiotypes included only a few strains (1–3 strains), there were 4 major antibiotypes registered. The most prevalent phenotype was antibiotype 1 which included 39 strains resistant to all antibiotics tested, except colistin. Antibiotype 2 included 27 strains susceptible to ceftazidime & avibactam, imipenem, imipenem & relebactam, meropenem, colistin and amikacin, and resistant to all other tested antibiotics. Antibiotype 3 included 22 strains which were susceptible to ceftazidime & avibactam, meropenem, colistin and amikacin, and antibiotype 4 was the most alarming one which included 19 strains that were resistant to all tested antibiotics, including colistin (Table 8). Pan-drug resistance (PDR) is defined as non-susceptibility to all antimicrobial agents in all antimicrobial categories that are available for use against a given microorganism [6]. Although our 19 strains are most likely PDR, they have not been tested against certain last-line agents such as cefiderocol, ceftobiprole, doripenem, meropenem-vaborbactam, and aztreonam-avibactam.

#### 2.4.2. Antibiotic Resistance Patterns of *E. coli* Involved in Colonization and Infection

A total of 60 clinical isolates of *E. coli* were tested for antimicrobial susceptibility. The results revealed a high prevalence of resistance, particularly to β-lactams and cephalosporins. All isolates (100%) were resistant to ampicillin, ceftriaxone, cefuroxime, cefotaxime, and ceftaroline. *E. coli* showed high rates of susceptibility among β-lactam/β-lactamase inhibitor combinations like piperacillin & tazobactam (78.3% strains susceptible), ceftazidime & avibactam (98.3% strains susceptible) and ceftolozane & tazobactam (85% strains susceptible). Meropenem remained highly effective, while resistance to ertapenem, imipenem, and imipenem & relebactam was high. Among non-β-lactams, colistin, tigecycline, and amikacin showed excellent activity, whereas gentamicin and tobramycin were moderately effective (Table 9). Both *E. coli* strains isolated from infections were resistant to ampicillin, ampicillin & sulbactam, amoxicillin & clavulanic acid, several cephalosporins (ceftriaxone, cefotaxime, cefuroxime, ceftaroline), and trimethoprim & sulfamethoxazole. They retained susceptibility to all other antibiotics tested. The 62 strains of *E. coli* were classified into 35 different antibiotypes, with no single antibiotype predominating. The MAR index for *E. coli* strains ranged from 0.32 to 0.8. Lower values (0.32–0.4) were seen in only a few isolates (3–4 each), while the most common value was 0.56 (15 isolates). Most strains clustered between 0.52 and 0.68. together representing the majority of strains. Only a small number exceeded 0.7 (*n* = 7), with no strains above 0.8.

#### 2.4.3. Antibiotic Resistance Patterns of Other *Enterobacterales* Involved in Colonization

*Enterobacter* spp. showed high resistance to many antibiotics, including ceftriaxone, cefotaxime, ceftazidime, ceftaroline, and trimethoprim & sulfamethoxazole (100% resistant). Most strains remained susceptible to meropenem (93.3%), ceftazidime & avibactam (93.3%) and amikacin (93.3%). All strains were susceptible to colistin (100%). *Enterobacter* spp. showed high percentage of resistance to ciprofloxacin (86.7%), but good susceptibility to levofloxacin (86.7%), indicating levofloxacin may be a more effective fluoroquinolone choice in this group (Table S7).

*Citrobacter* spp. showed mixed susceptibility. For some antibiotics (e.g., cefepime, carbapenems, colistin, amikacin), *Citrobacter* showed 100% susceptibility while for others like ceftriaxone, ceftazidime, and ciprofloxacin showed complete resistance or only 50% susceptibility. *S. marcescens* was highly resistant, with 100% resistance to most antibiotics tested, except for ceftazidime & avibactam, carbapenems, and amikacin, which showed full susceptibility. *Proteus* spp. showed high resistance to many antibiotics, including ampicillin, ceftriaxone, cefotaxime, ciprofloxacin, levofloxacin, trimethoprim & sulfamethoxazole, and ceftaroline (94–100% resistance). However, they were highly susceptible to piperacillin & tazobactam, ceftazidime & avibactam, meropenem, aztreonam, and amikacin (≥94% susceptible). Carbapenem susceptibility varied: meropenem was effective (94.1%), while ertapenem (70.6% resistant) and imipenem (64.7% resistant) were less so (Table S8).

*P. stuartii* exhibited resistance to all cephalosporins, carbapenems such as ertapenem and imipenem, fluoroquinolones, and aminoglycosides such as gentamicin and tobramycin, but they were susceptible to β-lactam/β-lactamase inhibitor combinations such as piperacillin & tazobactam, ceftazidime & avibactam, ceftolozane & tazobactam, meropenem, aztreonam, amikacin and trimethoprim & sulfamethoxazole. *M. morganii* showed high percentage of resistance to 3rd generation cephalosporins, carbapenems such as ertapenem and imipenem, fluoroquinolones and trimethoprim & sulfamethoxazole (66.7–100%), and they were susceptible to β-lactam/β-lactamase inhibitors combinations, cefepime, meropenem and aminoglycosides.

### 2.5. Antibiotic Resistance Patterns of Non-Fermentative Gram-Negative Bacilli

#### 2.5.1. Antibiotic Resistance Patterns of *P. aeruginosa* Involved in Colonization and Infection

*P. aeruginosa* isolates exhibited a high resistance to piperacillin & tazobactam, ceftazidime, cefepime, and carbapenems, with partial activity retained by ceftazidime & avibactam, ceftolozane & tazobactam, and amikacin. Fluoroquinolone resistance was nearly universal. Colistin was the only agent to retain complete activity, with 100% of isolates classified as susceptible (Table 10).

*P. aeruginosa* strains isolated from infections exhibited high rates of resistance to most tested antibiotics. All isolates were resistant to imipenem, imipenem & relebactam, ciprofloxacin, and levofloxacin (100%), with high resistance also seen to ceftazidime (86.7%), meropenem (93.3%), and piperacillin & tazobactam (80%). Partial susceptibility was observed for amikacin (46.7%) and aztreonam (40%). Other agents like cefepime (33.3% strains susceptible), ceftazidime & avibactam (33.3% strains susceptible), and ceftolozane & tazobactam (26.7% strains susceptible) showed limited activity. All isolates were susceptible to colistin (100%) (Table 11). The MAR index for *P. aeruginosa* strains ranged from 0.21 to 0.92. Only two strains were found at the lowest values (0.21 and 0.28). High resistance was frequent, with 21 strains at 0.85 and 14 at 0.92, making up the majority of the collection.

The 71 strains of *P. aeruginosa* were divided into 27 distinct antibiotypes, with 4 antibiotypes predominating. The most prevalent phenotype was antibiotype 1 which included 14 strains resistant to all tested antibiotics, except colistin, followed by antibiotype 2 with 11 strains, resistant to all tested antibiotics, except colistin and aztreonam. The third most prevalent phenotype included 9 strains which were resistant to all tested antibiotics, except colistin and amikacin, and the fourth most prevalent one included only 5 strains with susceptibility to ceftazidime, cefepime, ceftazidime & avibactam, ceftolozane & tazobactam, aztreonam, colistin and amikacin (Table 12). All other phenotypes were less frequent with only 1–3 strains present in each.

#### 2.5.2. Antibiotic Resistance Patterns of *A. baumannii* Involved in Colonization and Infection

All isolates (100%) were resistant to carbapenems, as well as to fluoroquinolones, indicating complete resistance to these commonly used therapeutic options. Susceptibility to aminoglycosides and trimethoprim & sulfamethoxazole was also extremely low. Colistin was the only agent to retain full in vitro activity, with 100% of isolates classified as susceptible (Table 13). All 23 strains isolated from infections presented with the same susceptibility pattern: they were susceptible only to colistin (100%), and they were resistant to all other tested antibiotics. For *A. baumannii*, 6 antibiotypes were identified, but the majority of strains (160 strains) were part of the same antibiotype, which presented with resistance to all tested antibiotics, except colistin. The MAR index of *A. baumannii* ranged from 0.66 to 0.88. Almost all isolates (*n* = 160), had a MAR index of 0.88, which demonstrates an overwhelming predominance of resistance among *A. baumannii* isolates.

#### 2.5.3. Antibiotic Resistance Patterns of *S. maltophilia* Involved in Colonization

Twelve clinical isolates of *S. maltophilia* were tested for susceptibility to Trimethoprim & Sulfamethoxazole (T/S). No isolates were fully susceptible according to EUCAST criteria. However, 66.7% (*n* = 8) were categorized as susceptible to increased exposure, indicating potential treatability with optimized dosing regimens. The remaining 33.3% (*n* = 4) were classified as resistant.

### 2.6. Antibiotic Resistance Patterns of Gram-Positive Bacteria

#### 2.6.1. Antibiotic Resistance Patterns of *Enterococcus* spp. Involved in Colonization

All strains of *E. faecium* (100%) were resistant to ampicillin, confirming the expected intrinsic resistance of this species. High rates of resistance were also observed to glycopeptides. Among last-resort agents, tigecycline retained full activity, with 100% susceptibility, while linezolid remained effective against most isolates. High-level aminoglycoside resistance (HLAR) was common, with 67.2% of isolates resistant to streptomycin and 86.2% to gentamicin. Only a minority lacked HLAR and may thus remain susceptible to combination therapy with cell-wall–active agents (Table 14).

All 58 strains of *E. faecium* were divided into 7 antibiotypes, with only 3 predominant antibiotypes. The majority of strains (34 strains) were part of antibiotype 1 which showed resistance to ampicillin, vancomycin, teicoplanin, streptomycin and gentamicin high-level resistance, and they were susceptible to tigecycline and linezolid. Antibiotype 2 included 12 strains with resistance to ampicillin, vancomycin, teicoplanin, gentamicin high-level resistance, but no streptomycin high-level resistance, and the third antibiotype included 6 strains with resistance to ampicillin, vancomycin, teicoplanin, but no streptomycin and gentamicin high-level resistance (Table 15). The MAR index for *E. faecium* ranged from 0.28 to 0.85. The majority of isolates recorded a MAR index of 0.57 (*n* = 13) and 0.71 (*n* = 34).

All strains of *E. faecalis* were fully susceptible to ampicillin, tigecycline, and linezolid (100%). Susceptibility to glycopeptides was observed in 83.3% (*n* = 5) of isolates for both vancomycin and teicoplanin, with one isolate (16.7%) demonstrating resistance to each agent. Regarding high-level aminoglycoside resistance (HLAR), high-level streptomycin resistance was detected in 66.7% (*n* = 4) of isolates, while high-level gentamicin resistance was identified in 83.3% (*n* = 5). Only 2 and 1 isolates, respectively, tested negative for HLAR (Table 16).

#### 2.6.2. Antibiotic Resistance Patterns of *S. aureus* Involved in Colonization and Infection

All strains of *S. aureus* were resistant to penicillin and oxacillin (100%), indicating that the entire collection consisted of methicillin-resistant *S. aureus* (MRSA) strains. Despite this resistance profile, several agents retained high or complete activity to most tested antibiotics. Complete resistance was observed for ciprofloxacin, levofloxacin, erythromycin, and tetracycline. High resistance rates were observed for linezolid and doxycycline (Table 17). The two strains of *S. aureus* isolated from infections showed resistance to penicillin, oxacillin, erythromycin, clindamycin, doxycycline, tetracycline, minocycline, trimethoprim & sulfamethoxazole, linezolid and rifampicin, and they were susceptible to all other antibiotics tested. For *S. aureus* there were 10 antibiotypes identified, with no single antibiotype predominating. The MAR index for *S. aureus* ranged from 0.21 to 0.43, with most isolates at the lower end of the scale (10 strains at 0.26, and 5 strains at 0.21). *S. aureus* isolates demonstrated relatively low MAR indices compared with the other species studied.

## 3. Discussion

This study provides an evaluation of bacterial colonization and subsequent healthcare-associated infections among ICU patients in a tertiary care hospital from Northeastern Romania, with a particular focus on MDR bacteria and their antibiotic resistance patterns. A total of 609 strains were isolated from 118 patients, highlighting an important microbial burden in this category of high-risk patients. Our study highlighted a predominance of Gram-negative bacilli involved in both colonization and infection and a poor representation of Gram-positive bacteria which may indicate a reduced pathogenic role in our cohort. The predominance of Gram-negative bacilli in both colonization and infection likely reflects the combined effects of antibiotic selection pressure, which favors MDR Gram-negative strains, and the ecological advantage of these organisms in the hospital environment, where they persist on equipment and in the gastrointestinal tract. Their numerous virulence factors may also facilitate progression from colonization to infection, explaining their overrepresentation among infecting strains. In contrast, the lower proportion of Gram-positive bacteria may suggest a reduced pathogenic role in this cohort, possibly due to stricter infection control measures or a lower adaptation to the hospital environment in this clinical context. These findings align with other studies across several geographical regions which also showed a predominant colonization of ICU patients with Gram-negative bacteria [19,20,21,22,23]. *A. baumannii* was the predominant organism isolated from pharyngeal and nasal swabs, whereas *K. pneumoniae*, *E. coli*, and *E. faecium* were more frequently found in rectal swabs. The preferential colonization of different anatomical sites reflects the ecological adaptation of the organisms. *K. pneumoniae*, *E. coli*, and *E. faecium* are enteric bacteria and therefore predominate in rectal swabs, whereas *A. baumannii* shows a greater affinity for pharyngeal and nasal mucosa, where it can survive and adhere to epithelial cells. These site-specific patterns are clinically relevant, as colonization of the upper airways by *A. baumannii* and other Gram-negative bacilli may serve as an important reservoir for subsequent lower respiratory tract infections, consistent with our finding of a strong association between pharyngeal/nasal colonization and respiratory infection. This significant association between pharyngeal/nasal colonization and subsequent lower respiratory tract infections was also demonstrated in other studies [24,25], while Huang et al. showed no relationship between respiratory colonization and subsequent development of respiratory tract infections [26]. However, in our study there was a higher prevalence of *A. baumannii* colonization and combined with poorer infection control measures can explain the discrepancies observed between the studies. Furthermore, *K. pneumoniae* and *A. baumannii* were among the most frequently isolated organisms on ICU admission, which may suggest that some patients were colonized before admission. This finding may reflect previous healthcare contact or the growing dissemination of MDR pathogens within the community. Although follow-up surveillance beyond day 7 was limited, our findings provide important insights into colonization dynamics in ICU patients. All 42 patients with extended follow-up acquired at least one additional MDR bacteria during their ICU stay, highlighting the rapid and ongoing risk of colonization in this setting. Moreover, the two patients that were screened at 28 days after ICU admission carried a total of 18 colonizing strains, and although the number may appear unusually high, both patients had a long period of ICU stay which was associated with prolonged exposure to invasive procedures, broad-spectrum antibiotics, and a high-risk environment for cross-transmission of MDR organisms. Extended ICU stays are known to promote colonization by multiple bacterial species due to selective antibiotic pressure and frequent contact with healthcare personnel and medical devices. Therefore, the large number of strains isolated from these two patients likely reflects the cumulative impact of prolonged hospitalization and intensive medical care. *A. baumannii* and *K. pneumoniae* remained the predominant colonizers, with *P. aeruginosa* consistently present at later time points. The persistence of these bacteria over time, even if the number of patients under surveillance decreased, suggests that they are well adapted to the ICU environment, potentially due to their intrinsic ability to survive on surfaces, form biofilms, and resist antibiotic pressure. These findings align with other studies demonstrating the ability of these bacteria to persist in ICU environments and act as reservoirs for infection [27,28,29]. The repeated acquisition of new MDR bacteria also indicates ongoing cross-transmission or endogenous expansion of resistant strains during prolonged ICU stay. Environmental contamination and selective antibiotic pressure have been identified as key drivers for MDR bacteria acquisition, particularly for *A. baumannii* and *P. aeruginosa* [28,30]. Clinically, these findings underscore the need for continued surveillance throughout the ICU stay and reinforce the importance of strict infection control practices, including environmental cleaning, hand hygiene, and targeted antimicrobial stewardship, to prevent colonization from progressing to infection and limit the spread of high-risk pathogens.

From the antibiotype point of view, we observed a high phenotypic diversity among colonizing isolates. Among *K. pneumoniae* strains, a high degree of phenotypic resistance diversity was observed which indicate a widespread circulation of various strains within the population. While most antibiotypes were represented by only 1–3 strains, four major antibiotypes accounted for the majority of isolates. The distribution of these antibiotypes revealed distinct epidemiological and clinical patterns with important implications for infection control and patient management.

The most prevalent profile (antibiotype 1) corresponded to an XDR phenotype resistant to all tested antibiotics except colistin. Its predominance in colonization, with only a few cases progressing to infection, highlights the role of colonized patients as reservoirs. The detection of these strains both at ICU admission and during ICU stay points to a dual origin: importation from external healthcare settings and ongoing nosocomial spread. This observation underscores the importance of admission screening and rigorous in-hospital infection prevention, particularly since prior studies showed that colonization with XDR bacteria substantially increases the risk of subsequent infection in critically ill patients [31,32]. Antibiotype 2 exhibited a more favorable susceptibility profile, and unlike antibiotype 1, this phenotype was more often acquired during ICU stay, suggesting that antibiotic selective pressure within the hospital environment contribute to its emergence [33]. Although predominantly associated with colonization, its involvement in infections, especially those linked to prior colonization, underlines its clinical significance, however considering the broad therapeutic options available for this phenotype, infections caused by this antibiotype may be more manageable if they are appropriately treated. Antibiotype 3 was associated only with colonization and had a higher prevalence at ICU admission which suggests that its often imported rather than acquired during ICU stay. This finding emphasizes the importance of screening upon ICU admission, since colonized patients may serve as sources for cross-transmission even if they are not at immediate risk of infection [34]. While less clinically alarming than antibiotypes 1 and 4, its potential to evolve under antimicrobial pressure remains concerning, especially given the limited therapeutic options available in the ICU. Finally, antibiotype 4 represented the most concerning phenotype, displaying resistance to all tested antibiotics, including colistin. Its involvement in both colonization and infection, and both its presence upon ICU admission and its acquisition during ICU stay highlights the dual challenge of external introduction and nosocomial dissemination. The clinical implications are particularly severe, as infections with such strains are essentially untreatable, a problem well documented in recent reports of infections with colistin-resistant *K. pneumoniae* [35,36]. The emergence of colistin resistance was also reported in recent surveillance data from countries such as Romania, Greece and Italy reporting elevated rates (25.8%, 19.9% and 15.4%, respectively) [15,16,17,18]. Another study from Northeastern Romania also showed that *K. pneumoniae* exhibited a high prevalence of colistin resistance over the last 5 years, with annual resistance rates fluctuating between 12.97% and 21.64% [37]. The presence of this phenotype emphasizes the urgent need for strict infection control measures, active surveillance, and strict antimicrobial stewardship to limit the spread of these high-risk clones.

For *E. coli*, we identified a considerable phenotypic diversity in antibiotic resistance. No predominant antibiotypes were identified, with many antibiotypes being represented by 1–2 strains which implies high variability in antibiotic resistance. Notably, only one strain of *E. coli* was not present at ICU admission and was acquired during ICU stay, suggesting a limited cross-transmission of *E. coli* in ICU, being more often introduced from external sources. However, the two strains involved in infections were also involved in colonization and had the same antibiotype, highlighting the role of colonization as a precursor to infection. These findings underscore the importance of screening to identify patients at risk, support targeted antimicrobial therapy based on susceptibility profiles, and suggest that infection control measures are effective in limiting in-ICU transmission. Other studies highlighted the complexity of *E. coli* colonization, its phenotypic diversity, and the dynamics of infection in ICU settings, emphasizing the importance of genomic surveillance to establish the complexity of *E. coli* colonization and the potential for strain evolution within ICU settings [38,39].

For *A. baumannii,* we observed a marked predominance of a single phenotype which was characterized by resistance to all tested antibiotics except colistin. This highlights the therapeutic limitations clinicians face, as colistin remains one of the last active agents against XDR *A. baumannii*. Similar findings regarding increased rates of resistance and an alarming reliance on colistin for effective therapy have been reported [40,41,42]. The clinical relevance of this finding is underscored by the fact that all strains involved in infections were part of this antibiotype and moreover, all strains involved in infections were also involved in colonization suggesting a strong correlation between colonization and infection with *A. baumannii*. This finding aligns with previous studies demonstrating that colonization is a prerequisite for *A. baumannii* infection, particularly in ICU patients [24,43]. Out of the 137 strains involved in colonization, 44 were acquired during ICU stay, which emphasize that cross-transmission within the ICU is concerning. This is consistent with outbreak investigations demonstrating that clonal spread within the ICU occurs via contaminated surfaces, shared equipment, and healthcare worker hands. *A. baumannii* is well known for its ability to survive desiccation and persist on inanimate surfaces, facilitating environmental reservoirs that sustain transmission [44,45,46].

Regarding *P. aeruginosa* strains, our study revealed a marked phenotypic diversity. Colonization was more common than infection, consistent with the opportunistic nature of this pathogen in ICU settings, also stated by Harris et al. [47]. The predominance of a few major antibiotypes highlights the emergence of highly resistant clones. Notably, antibiotype 1, resistant to all antibiotics except colistin, was the most frequent and concerning, indicating limited therapeutic options although most isolates were associated with colonization. Antibiotypes 2 and 3, also showing extensive resistance, were exclusively acquired during ICU stay, suggesting in-hospital selection pressures and possible cross-transmission. This pattern highlights the critical role of the ICU environment in driving the emergence of MDR *P. aeruginosa* and the need for strict infection control measures. In contrast, antibiotype 4 displayed broader susceptibility and a lower prevalence, indicating that less resistant strains persist but are less competitive in the hospital environment. Interestingly, all patients who developed an infection with *P. aeruginosa* were colonized with the same pathogen, however the antibiotypes did not match in most cases. Out of the 15 strains involved in infections, 9 strains had a different antibiotype than the one involved in colonization. This finding may suggest that the infecting strain may have replaced the initial colonizing strain during the ICU stay, reflecting dynamic shifts in the patient’s microbial flora under selective pressure from antibiotics on environmental exposure, or horizontal gene transfer or rapid mutational events may have altered the resistance phenotype between colonization and infection, particularly under strong antimicrobial pressure. Several studies observed the mismatch between colonizing and infecting *P. aeruginosa* antibiotypes [48,49,50,51,52]. One possibility for this mismatch is strain replacement, where new, often more resistant strains displace resident colonizing populations under antibiotic selective pressure [49]. Within-host diversity can also accelerate resistance evolution. For example, patients colonized by multiple strains tend to select for pre-existing resistant strains, while patients with single-strain colonization acquire resistance more sporadically through novel mutations. Additionally, within-host evolution generates phenotypic diversity, enabling initially colonizing strains to acquire resistance mutations or virulence traits that facilitate transition to infection [49,50]. Environmental and patient factors, such as immune status and ICU exposure, further influence which strains persist and which cause infection [51,52]. Clinically, these dynamics have several implications. The divergence between colonizing and infecting strains complicates empiric therapy, as the antibiotic susceptibility of colonizing strains may not reliably predict the susceptibility profile of infecting strains. This underscores the need for continuous surveillance and molecular typing to detect emergent resistant clones and guide antimicrobial stewardship [51]. Also, the potential for rapid evolution and strain mixing highlights the importance of strict infection control measures, including grouping patients colonized or infected with the same pathogen together, environmental decontamination, and hand hygiene, to limit in-hospital transmission [49]. Recognizing colonization as a reservoir for MDR *P. aeruginosa* can therefore inform both preventive strategies and treatment decisions, ultimately reducing ICU morbidity and mortality. Another notable finding regarding *P. aeruginosa* was the very high rates of resistance to carbapenems, compared to cephalosporins such as ceftazidime and cefepime, where resistance rates were somewhat lower. Mechanisms for carbapenem resistance likely result from the loss of *OprD* porin, efflux pump overexpression, and carbapenemase production, which offers high-level, often complete resistance to carbapenems, while for cephalosporins, resistance is offered by an overexpression of *AmpC* β-lactamase or altered membrane permeability, which reduce but do not fully eliminate drug activity. This can explain why cephalosporins showed more intermediate results than carbapenems, despite both being β-lactam agents [53,54,55].

All isolates (100%) of both *P. aeruginosa* and *A. baumannii* were classified as susceptible to colistin, which emphasizes the critical role of this last-line antibiotic in treating infections caused by non-fermentative Gram-negative bacilli. Although in our study no strain was resistant to colistin, others reported resistant strains of *P. aeruginosa* and *A. baumannii* [56,57,58] emphasizing the need for development of new agents for these critical pathogens.

When looking at resistance patterns for Gram-positive bacteria, we observed the same alarming resistance profiles observed in Gram-negative bacteria. For *Enterococcus* spp. we observed key differences between the two species. All *E. faecium* isolates displayed a very high resistance to both vancomycin and teicoplanin, but a low resistance to linezolid and no resistance to tigecycline, thus preserving some treatment options. HLAR was frequent, significantly limiting the potential for effective synergistic combination therapy. In contrast, all *E. faecalis* isolates remained susceptible to ampicillin, tigecycline, and linezolid. Glycopeptide resistance was less common, with only one isolate resistant to vancomycin and teicoplanin, suggesting sporadic occurrence of VRE in this species. All isolates of *S. aureus* were methicillin-resistant (MRSA), with 100% resistance to both penicillin and oxacillin, however all isolates were susceptible to anti-MRSA agents. Notably, linezolid resistance was detected in 63.2% of isolates which is an alarming finding compared to other studies who showed very low rates of resistance to linezolid (<5%) [59,60]. Several factors may explain these findings, including local clonal expansion or outbreak of resistant MRSA strains, selective pressure from prior use of linezolid as a therapeutic choice, and horizontal transfer of resistance genes such as *cfr* or *optrA* [61]. Patient-related risk factors such as prolonged ICU stay or invasive devices, and potential lapses in infection control may have further contributed. These observations underscore the importance of enhanced surveillance, molecular typing to track resistant clones, strict infection prevention measures, and antimicrobial stewardship to mitigate the emergence and spread of linezolid-resistant MRSA in critical care settings. VRE and MRSA remain important challenges in ICU settings, considering the high prevalence of MDR profiles reported by several studies [21,23,62,63].

Recent studies conducted in Romanian ICUs also highlight the high burden and evolving dynamics of antimicrobial resistance among Gram-negative pathogens. Golli et al. (2019) reported high MDR rates for several pathogens, such as *P. aeruginosa* (64.7%), MRSA (62.7%), and *Klebsiella* spp. (53.3%) [64]. Also, they highlighted the impact of the COVID-19 pandemic, showing a post-pandemic rise in MDR and PDR strains, particularly among *Klebsiella* and *Acinetobacter*, including resistance to last-resort agents such as colistin and tigecycline [65,66]. Another study by Apetroaei et al. (2025) demonstrated that *A. baumannii* and *P. aeruginosa* continue to display the highest resistance levels, particularly in high-risk units such as the ICU and transplant wards [67]. Collectively, these findings underscore the persistent and evolving threat of MDR and PDR Gram-negative pathogens in ICU settings, emphasizing the urgent need for continuous surveillance and robust antimicrobial stewardship strategies to mitigate the spread of these high-risk organisms [64,65,66,67]. Finally, when looking at the MAR index for all bacterial strains included in our study we observed an alarming trend. A MAR index > 0.2 indicates that the bacteria come from a high-risk source where antibiotics are frequently used, while a MAR index ≤ 0.2 indicates that the isolate is from an environment with infrequent or less intensive antibiotic exposure. In our study, all strains had a MAR index > 0.2, with the majority of strains having a MAR index > 0.8. This suggests that every strain originated from high-risk environments with frequent antibiotic exposure, such as hospital wards, especially ICUs, where antimicrobial use is intensive. MAR values exceeding 0.8 suggest repeated or prolonged exposure to diverse antimicrobials, allowing resistant clones to thrive and spread. Others showed that MAR values of bacterial strains isolated from ICUs tend to be higher than those isolated from surgical/medical wards [68].

Our study has several limitations regarding dynamics between colonization and infection. Only 42 patients were followed-up beyond ICU admission, with a decreasing number of patients screened at later time points, which may have led to underestimation of late-onset colonization or infection. Also, information regarding patients’ prior hospitalizations, antibiotic exposure, or invasive medical procedures was not available, so it is plausible that some patients were colonized before admission. Further studies should incorporate detailed patient history to more accurately distinguish between community and healthcare associated colonization. Another key limitation is the absence of molecular typing methods to confirm the relatedness of isolates involved in colonization and infection. Although we used antibiotypes to assess the correlations between colonization and infection, future studies integrating genotypic methods are needed to provide more robust insights into transmission dynamics and strain relatedness.

## 4. Materials and Methods

### 4.1. Study Design and Population

We conducted a prospective observational study to investigate the relationship between colonization and subsequent infection with MDR bacteria in the ICU of a tertiary care hospital in Iași, Romania. The study was carried out at the Clinical Hospital of Infectious Diseases “Sf. Parascheva” between January and October 2024. We included adult patients (≥18 years of age) who were admitted to the ICU and found to be colonized with MDR bacteria during their hospital stay. Colonization was identified through active surveillance cultures collected systematically, and it was defined as the presence of the targeted microorganisms without evidence of tissue invasion or inflammation at that body site [69]. All patients > 18 years old who had a documented colonization with MDR bacteria upon ICU admission and had a longer ICU stay than 48 h were included in the study. Patients with no documented colonization at ICU admission but who acquired at least one MDR bacteria during ICU stay were also included in the study. Patients were excluded if they were under 18 years of age, had an ICU stay of less than 48 h, and had no documented colonization during ICU stay. Surveillance cultures were obtained from each patient upon admission to the ICU and subsequently every seven days during the ICU stay. For each time point, three samples were collected (nasal swab, pharyngeal swab, and rectal swab) to detect colonization with selected MDR pathogens. The samples were collected in accordance with the laboratory’s pathological sample collection manual. The swabs were rotated at least 5 times at the collection site, in order to ensure proper absorption of the biological material by the swab. Multiple isolates per patient were included if they were obtained from different sites or time points. The targeted organisms included methicillin-resistant *Staphylococcus aureus* (MRSA), extended-spectrum beta-lactamase (ESBL)-producing *Enterobacterales*, carbapenem-resistant Gram-negative bacilli, and vancomycin-resistant enterococci (VRE). In cases where patients enrolled in this study developed infections during their stay in the ICU, the bacterial strains associated with these infections were also collected and included in the analysis. Infection was defined as the invasion of the body tissues by the pathogens, resulting in disease [69].

### 4.2. Isolation and Identification of Bacteria

Isolation of MDR bacteria from nasal, pharyngeal and rectal swabs was performed on chromogenic media for MDR bacteria (Thermo Fisher Scientific, Oxoid Ltd., Basingstoke, UK). Chromogenic media were incubated at 35 ± 2 °C and bacterial growth was assessed after 24 and 48 h of incubation. Bacteria involved in infections were isolated from tracheal aspirates, blood and urine, and they were isolated on standard media recommended for each pathological sample. All bacteria were identified using matrix-assisted laser desorption ionization-time-of-flight mass spectrometry (MALDI-TOF MS) (Bruker, Bremen, Germany).

### 4.3. Antimicrobial Susceptibility Testing

Antibiotic susceptibility testing was performed using the broth microdilution method in an automated system (MICRONAUT-S). Antibiotic susceptibility testing for Gram-negative bacteria was performed using MICRONAUT-S Romania GN 2 EUCAST panels (Bruker, Bremen, Germany) and for Gram-positive bacteria MICRONAUT-S Romania GP 3 EUCAST panels (Bruker, Bremen, Germany) were used. Testing was performed according to instructions provided by the producer. After incubation, the minimum inhibitory concentrations (MICs) were visually read and interpretation of the results was performed according to the European Committee on Antimicrobial Susceptibility Testing (EUCAST) standard [70]. For quality control, standard reference strains such as *Escherichia coli* ATCC 25922, *Pseudomonas aeruginosa* ATCC 27853, *Staphylococcus aureus* ATCC 29213 and *Enterococcus faecalis* ATCC 29212 were included in each testing series.

### 4.4. Statistical Analysis

Results were analyzed using the 25th version of the IBM SPSS statistical software. Associations between colonization and infection were assessed using the Chi-square test. A *p*-value of <0.05 was considered indicative of statistical significance.

### 4.5. Ethical Principles

The study complied with the ethical principles stated by the World Medical Association’s Declaration of Helsinki regarding medical research involving human subjects. The study was approved by the Ethics Committee of the University of Medicine and Pharmacy “Grigore T. Popa”, Iași, Romania (IRB No. 458/26 June 2024), as well as from the Ethics Committee of the Clinical Hospital of Infectious Diseases “Sf. Parascheva”, Iași, Romania (IRB No. 4/20 March 2024). Informed consent was obtained from all subjects involved in the study.

## 5. Conclusions

This study demonstrates a high colonization-to-infection link among ICU patients in a Northeastern Romanian tertiary care hospital, providing novel evidence from a region with limited published data. These findings underscore the importance of systematic colonization screening to identify patients at high risk in ICU settings. Strengthening infection prevention and control measures, together with reinforcing antimicrobial stewardship programs, is essential to limit transmission of MDR pathogens and preserve therapeutic options in the ICU.

## Figures and Tables

**Table 1:** Distribution of bacterial strains involved in colonization according to screening time points.

	Number of Patients Screened	Number of Bacterial Strains Isolated from Pharyngeal Exudate	Number of Bacterial Strains Isolated from Nasal Exudate	Number of Bacterial Strains Isolated from Rectal Swabs	Total Number of Bacterial Strains
ICU admission	118	83	36	163	282
7 days after ICU admission	42	61	26	65	152
14 days after ICU admission	14	25	18	16	59
21 days after ICU admission	8	14	7	8	29
28 days after ICU admission	2	7	3	8	18
35 days after ICU admission	1	3	3	2	8
Total	118	193	93	262	548

**Table 2:** Distribution of bacterial strains according to colonization and infection.

	Total Number of Bacterial Strains		Gram-Negative Bacteria		Gram-Positive Bacteria	
Colonization	548	89.98%	465	84.85%	83	15.15%
Infection	61	10.02%	59	96.72%	2	3.28%

**Table 3:** Distribution of bacterial strains according to pathological sample.

	Pathological Sample	Number of Bacterial Strains	
Colonization	Pharyngeal exudate	193	35.22%
	Nasal exudate	93	16.97%
	Rectal swab	262	47.81%
	Total	548	100%
Infection	Tracheal aspirate	31	50.82%
	Urine	15	24.59%
	Blood	13	21.31%
	Sputum	1	1.64%
	Conjunctival secretion	1	1.64%
	Total	61	100%

**Table 4:** Distribution of bacteria involved in infections.

	Lower Respiratory Tract Infections		Urinary Tract Infections		Bloodstream Infections		Conjunctivitis		Total
	n	%	n	%	n	%	n	%	n (%)
*Acinetobacter baumannii*	17	73.9%	2	8.7%	4	17.4%	-	-	23 (100%)
*Klebsiella pneumoniae*	4	21.1%	10	52.6%	5	26.3%	-	-	19 (100%)
*Pseudomonas aeruginosa*	10	66.6%	3	20%	1	6.7%	1	6.7%	15 (100%)
*Escherichia coli*	1	50%	-	-	1	50%	-	-	2 (100%)
*Staphylococcus aureus*	-	-	-	-	2	100%	-	-	2 (100%)

**Table 5:** Distribution of antibiotypes involved in infection and/or colonization.

Bacteria	AMP	AMS	AMC	PIT	CRO	CXM	CAA	CTX	CAZ	CTA	CFL	CEP	ERT	IMP	IMR	MER	AZT	COL	CIP	LEV	T/S	AMK	GEN	TOB	TGC	Infection	Colonization
*K. pneumoniae*																										5	5
																										4	3
																										4	2
																										2	-
																										1	-
																										1	-
																										1	-
																										1	-
*E. coli*																										2	1
*P. aeruginosa*																										3	-
																										3	3
																										2	-
																										1	1
																										1	1
																										1	-
																										1	1
																										1	-
																										1	-
																										1	-
*A. baumannii*																										23	22

AMP—ampicillin; AMS—ampicillin & sulbactam; AMC—amoxicillin & clavulanic acid; PIT—piperacillin & tazobactam; CRO—ceftriaxone; CXM—cefuroxime; CAA—ceftazidime & avibactam; CTX—cefotaxime; CAZ—ceftazidime; CTA—ceftolozane & tazobactam; CFL—ceftaroline; CEP—cefepime; ERT—ertapenem; IMP—imipenem; IMR—imipenem & relebactam; MER—meropenem; AZT—aztreonam; COL—colistin; CIP—ciprofloxacin; LEV—levofloxacin; T/S—trimethoprim & sulfametoxazole; AMK—amikacin; GEN—gentamicin; TOB—tobramycin; TGC—tigecycline; Green—susceptible, standard dosing; Yellow—susceptible, increased exposure; Red—resistant.

**Table 6:** Antibiotic resistance patterns of *K. pneumoniae* involved in colonization (146 strains).

Antibiotic	Susceptible, Standard Dosing (S)		Susceptible, Increased Exposure (I)		Resistant (R)	
	n	%	n	%	n	%
Ampicillin & Sulbactam	-	-	-	-	146	100%
Amoxicillin & Clavulanic acid	3	2.1%	-	-	143	97.9%
Piperacillin & Tazobactam	20	13.7%	-	-	126	86.3%
Cefuroxime	-	-	-	-	146	100%
Ceftriaxone	-	-	-	-	146	100%
Cefotaxime	-	-	-	-	146	100%
Ceftazidime	5	3.4%	7	4.8%	134	91.8%
Cefepime	-	-	5	3.4%	141	96.6%
Ceftaroline	-	-	-	-	146	100%
Ceftazidime & Avibactam	86	58.9%	-	-	60	41.1%
Ceftolozane & Tazobactam	27	18.5%	-	-	119	81.5%
Ertapenem	18	12.3%	-	-	128	87.7%
Imipenem	43	29.5%	1	0.7%	102	69.9%
Imipenem & Relebactam	44	30.1%	-	-	102	69.9%
Meropenem	74	50.7%	2	1.4%	70	47.9%
Aztreonam	10	6.8%	2	1.4%	134	91.8%
Ciprofloxacin	4	2.7%	6	4.1%	136	93.2%
Levofloxacin	14	9.6%	-	-	132	90.4%
Amikacin	86	58.9%	-	-	60	41.1%
Gentamicin	22	15.1%	-	-	124	84.9%
Tobramycin	11	7.5%	-	-	135	92.5%
Trimethoprim & Sulfamethoxazole	6	4.1%	-	-	140	95.9%
Colistin	125	85.6%	-	-	21	14.4%

**Table 7:** Antibiotic resistance patterns of *K. pneumoniae* involved in infection (19 strains).

Antibiotic	Susceptible, Standard Dosing (S)		Susceptible, Increased Exposure (I)		Resistant (R)	
	n	%	n	%	n	%
Ampicillin & Sulbactam	-	-	-	-	19	100%
Amoxicillin & Clavulanic acid	1	5.3%	-	-	18	94.7%
Piperacillin & Tazobactam	1	5.3%	-	-	18	94.7%
Cefuroxime	-	-	-	-	19	100%
Ceftriaxone	-	-	-	-	19	100%
Cefotaxime	-	-	-	-	19	100%
Ceftazidime	-	-	-	-	19	100%
Cefepime	-	-	-	-	19	100%
Ceftaroline	-	-	-	-	19	100%
Ceftazidime & Avibactam	9	47.4%	-	-	10	52.6%
Ceftolozane & Tazobactam	1	5.3%	-	-	18	94.7%
Ertapenem	-	-	-	-	19	100%
Imipenem	5	26.3%	-	-	14	73.7%
Imipenem & Relebactam	6	31.6%	-	-	13	68.4%
Meropenem	6	31.6%	1	5.3%	12	63.1%
Aztreonam	1	5.3%	-	-	18	94.7%
Ciprofloxacin	-	-	-	-	19	100%
Levofloxacin	-	-	-	-	19	100%
Amikacin	10	52.6%	-	-	9	47.4%
Gentamicin	1	5.3%	-	-	18	94.7%
Tobramycin	1	5.3%	-	-	18	94.7%
Trimethoprim & Sulfamethoxazole	-	-	-	-	19	100%
Colistin	13	68.4%	-	-	6	31.6%

**Table 8:** Classification of *K. pneumoniae* strains according to major antibiotypes.

Antibiotype	n	AMS	AMC	PIT	CRO	CXM	CAA	CTX	CAZ	CTA	CFL	CEP	ERT	IMP	IMR	MER	AZT	COL	CIP	LEV	T/S	AMK	GEN	TOB
Antibiotype 1	39																							
Antibiotype 2	27																							
Antibiotype 3	22																							
Antibiotype 4	19																							

AMS—ampicillin & sulbactam; AMC—amoxicillin & clavulanic acid; PIT—piperacillin & tazobactam; CRO—ceftriaxone; CXM—cefuroxime; CAA—ceftazidime & avibactam; CTX—cefotaxime; CAZ—ceftazidime; CTA—ceftolozane & tazobactam; CFL—ceftaroline; CEP—cefepime; ERT—ertapenem; IMP—imipenem; IMR—imipenem & relebactam; MER—meropenem; AZT—aztreonam; COL—colistin; CIP—ciprofloxacin; LEV—levofloxacin; T/S—trimethoprim & sulfametoxazole; AMK—amikacin; GEN—gentamicin; TOB—tobramycin; Green—susceptible, standard dosing; Red—resistant.

**Table 9:** Antibiotic resistance patterns of *E. coli* involved in colonization (60 strains).

Antibiotic	Susceptible, Standard Dosing (S)		Susceptible, Increased Exposure (I)		Resistant (R)	
	n	%	n	%	n	%
Ampicillin	-	-	-	-	60	100%
Ampicillin & Sulbactam	2	3.3%	-	-	58	96.7%
Amoxicillin & Clavulanic acid	15	25%	-	-	45	75%
Piperacillin & Tazobactam	47	78.3%	-	-	13	21.7%
Cefuroxime	-	-	-	-	60	100%
Ceftriaxone	-	-	-	-	60	100%
Cefotaxime	-	-	-	-	60	100%
Ceftazidime	3	5%	7	11.7%	50	83.3%
Cefepime	-	-	6	10%	54	90%
Ceftaroline	-	-	-	-	60	100%
Ceftazidime & Avibactam	59	98.3%	-	-	1	1.7%
Ceftolozane & Tazobactam	51	85%	-	-	9	15%
Ertapenem	33	55%	-	-	27	45%
Imipenem	33	55%	-	-	27	45%
Imipenem & Relebactam	33	55%	-	-	27	45%
Meropenem	58	96.7%	1	1.7%	1	1.7%
Aztreonam	6	10%	3	5%	51	85%
Ciprofloxacin	14	23.3%	3	5%	43	71.7%
Levofloxacin	20	33.3%	-	-	40	66.7%
Amikacin	59	98.3%	-	-	1	1.7%
Gentamicin	36	60%	-	-	24	40%
Tobramycin	32	53.3%	-	-	28	46.7%
Trimethoprim & Sulfamethoxazole	3	5%	1	1.7%	56	93.3%
Tigecycline	57	95%	-	-	3	5%
Colistin	60	100%	-	-	-	-

**Table 10:** Antibiotic resistance patterns of *P. aeruginosa* involved in colonization (56 strains).

Antibiotic	Susceptible, Standard Dosing (S)		Susceptible, Increased Exposure (I)		Resistant (R)	
	n	%	n	%	n	%
Piperacillin & Tazobactam	-	-	8	14.3%	48	85.7%
Ceftazidime	-	-	15	26.8%	41	73.2%
Cefepime	-	-	20	35.7%	36	64.3%
Ceftazidime & Avibactam	25	44.6%	-	-	31	55.4%
Ceftolozane & Tazobactam	21	37.5%	-	-	35	62.5%
Imipenem	-	-	1	1.8%	55	98.2%
Imipenem & Relebactam	3	5.4%	-	-	53	94.6%
Meropenem	2	3.6%	3	5.4%	51	91.1%
Aztreonam	-	-	30	53.6%	26	46.4%
Ciprofloxacin	-	-	-	-	56	100%
Levofloxacin	-	-	1	1.8%	55	98.2%
Amikacin	29	51.8%	-	-	27	48.2%
Tobramycin	1	1.8%	-	-	55	98.2%
Colistin	56	100%	-	-	-	-

**Table 11:** Antibiotic resistance patterns of *P. aeruginosa* involved in infection (15 strains).

Antibiotic	Susceptible, Standard Dosing (S)		Susceptible, Increased Exposure (I)		Resistant (R)	
	n	%	n	%	n	%
Piperacillin & Tazobactam	-	-	3	20%	12	80%
Ceftazidime	-	-	2	13.3%	13	86.7%
Cefepime	-	-	5	33.3%	10	66.7%
Ceftazidime & Avibactam	5	33.3%	-	-	10	66.7%
Ceftolozane & Tazobactam	4	26.7%	-	-	11	73.3%
Imipenem	-	-	-	-	15	100%
Imipenem & Relebactam	-	-	-	-	15	100%
Meropenem	-	-	1	6.7%	14	93.3%
Aztreonam	-	-	6	40%	9	60%
Ciprofloxacin	-	-	-	-	15	100%
Levofloxacin	-	-	-	-	15	100%
Amikacin	7	46.7%	-	-	8	53.3%
Tobramycin	1	6.7%	-	-	14	93.3%
Colistin	15	100%	-	-	-	-

**Table 12:** Classification of *P. aeruginosa* strains according to major antibiotypes.

Antibiotype	n	PIT	CAA	CAZ	CTA	CEP	IMP	IMR	MER	AZT	COL	CIP	LEV	AMK	TOB
Antibiotype 1	14														
Antibiotype 2	11														
Antibiotype 3	9														
Antibiotype 4	5														

PIT—piperacillin&tazobactam; CAA—ceftazidime&avibactam; CAZ—ceftazidime; CTA—ceftolozane&tazobactam; CEP—cefepime; IMP—imipenem; IMR—imipenem&relebactam; MER—meropenem; AZT—aztreonam; COL—colistin; CIP—ciprofloxacin; LEV—levofloxacin; AMK—amikacin; TOB—tobramycin; Green—susceptible, standard dosing; Yellow—susceptible, increased exposure; Red—resistant.

**Table 13:** Antibiotic resistance patterns of *A. baumannii* involved in colonization (146 strains).

Antibiotic	Susceptible, Standard Dosing (S)		Susceptible, Increased Exposure (I)		Resistant (R)	
	n	%	n	%	n	%
Imipenem	-	-	-	-	146	100%
Meropenem	-	-	-	-	146	100%
Ciprofloxacin	-	-	-	-	146	100%
Levofloxacin	-	-	-	-	146	100%
Amikacin	3	2.1%	-	-	143	97.9%
Gentamicin	4	2.7%	-	-	142	97.3%
Tobramycin	2	1.4%	-	-	144	98.6%
Trimethoprim & Sulfamethoxazole	2	1.4%	-	-	144	98.6%
Colistin	146	100%	-	-	-	-

**Table 14:** Antibiotic resistance patterns of *E. faecium* involved in colonization (58 strains).

Antibiotic	Susceptible, Standard Dosing (S)		Susceptible, Increased Exposure (I)		Resistant (R)		HLAR Negative	HLAR Positive
	n	%	n	%	n	%	n (%)	n (%)
Ampicillin	-	-	-	-	58	100%		
Vancomycin	2	3.4%	-	-	56	96.6%		
Teicoplanin	2	3.4%	-	-	56	96.6%		
Tigecycline	58	100%	-	-	-	-		
Linezolid	54	93.1%	-	-	4	6.9%		
Streptomycin high level	-	-	-	-	-	-	19 (32.8%)	39 (67.2%)
Gentamicin high level	-	-	-	-	-	-	8 (13.8%)	50 (86.2%)

**Table 15:** Classification of *E. faecium* strains according to major antibiotypes.

Antibiotype	Number	AMP	SNH	GNH	VAN	TPL	TGC	LIZ
Antibiotype 1	34	R	positive	positive	R	R	S	S
Antibiotype 2	12	R	negative	positive	R	R	S	S
Antibiotype 3	6	R	negative	negative	R	R	S	S

AMP—ampicillin; SNH—streptomycin high level; GNH—gentamicin high level; VAN—vancomycin; TPL—teicoplanin; TGC—tigecycline; LIZ—linezolid; S—susceptible, standard dosing; R—resistant.

**Table 16:** Antibiotic resistance patterns of *E. faecalis* involved in colonization (6 strains).

Antibiotic	Susceptible, Standard Dosing (S)		Susceptible, Increased Exposure (I)		Resistant (R)		HLAR Negative	HLAR Positive
	n	%	n	%	n	%	n (%)	n (%)
Ampicillin	6	100%	-	-	-	-		
Vancomycin	5	83.3%	-	-	1	16.7%		
Teicoplanin	5	83.3%	-	-	1	16.7%		
Tigecycline	6	100%	-	-	-	-		
Linezolid	6	100%	-	-	-	-		
Streptomycin high level	-	-	-	-	-	-	2 (33.3%)	4 (66.7%)
Gentamicin high level	-	-	-	-	-	-	1 (16.7%)	5 (83.3%)

**Table 17:** Antibiotic resistance patterns of *S. aureus* involved in colonization (19 strains).

Antibiotic	Susceptible, Standard Dosing (S)		Susceptible, Increased Exposure (I)		Resistant (R)	
	n	%	n	%	n	%
Penicillin	-	-	-	-	19	100%
Oxacillin	-	-	-	-	19	100%
Ceftaroline	19	100%	-	-	-	-
Ciprofloxacin	-	-	19	100%	-	-
Levofloxacin	-	-	19	100%	-	-
Moxifloxacin	19	100%	-	-	-	-
Amikacin	19	100%	-	-	-	-
Gentamicin	18	94.7%	-	-	1	5.3%
Tobramycin	16	84.2%	-	-	3	15.8%
Erythromycin	-	-	-	-	19	100%
Clindamycin	16	84.2%	-	-	3	15.8%
Vancomycin	19	100%	-	-	-	-
Teicoplanin	19	100%	-	-	-	-
Daptomycin	19	100%	-	-	-	-
Doxycycline	4	21.1%	-	-	15	78.9%
Minocycline	16	84.2%	-	-	3	15.8%
Tetracycline	-	-	-	-	19	100%
Tigecycline	19	100%	-	-	-	-
Trimethoprim & Sulfamethoxazole	18	94.7%	-	-	1	5.3%
Linezolid	7	36.8%	-	-	12	63.2%
Rifampicin	15	78.9%	-	-	4	21.1%
Fusidic acid	19	100%	-	-	-	-
Dalbavancin	19	100%	-	-	-	-

## Data Availability

The data are contained within the article and in Supplementary Tables S1–S8.

## Supplementary Materials

The following supporting information can be downloaded at: https://www.mdpi.com/article/10.3390/antibiotics14111063/s1, Table S1: Distribution of bacteria involved in colonization upon ICU admission (282 strains); Table S2: Distribution of bacteria involved in colonization at 7 days after ICU admission (152 strains); Table S3: Distribution of bacteria involved in colonization at 14 days after ICU admission (59 strains); Table S4: Distribution of bacteria involved in colonization at 21 days after ICU admission (29 strains); Table S5: Distribution of bacteria involved in colonization at 28 days after ICU admission (18 strains); Table S6: Distribution of bacteria involved in colonization at 35 days after ICU admission (8 strains); Table S7: Antibiotic resistance patterns of Enterobacter spp. involved in colonization (15 strains); Table S8: Antibiotic resistance patterns of Proteus spp. involved in colonization (17 strains).
