# Supplementary material for: Antibiotic Resistance Patterns of Bacteria Involved in Colonization and/or Infection of Patients in Intensive Care Units in Northeastern Romania
Source: Antibiotics (Basel). 2025 Oct 23;14(11):1063. doi: 10.3390/antibiotics14111063 (PMC12649633; doi:10.3390/antibiotics14111063)
Supplement: Supplementary file 1 [file antibiotics-14-01063-s001.zip › antibiotics-3853449-supplementary.pdf]

**Table S1.** Distribution of bacteria involved in colonization upon ICU admission (282 strains)

| | **Pharyngeal exudate** | | **Nasal exudate** | | **Rectal swab** | |
|---|---|---|---|---|---|---|
| | Number | Percentage | Number | Percentage | Number | Percentage |
| *Klebsiella pneumoniae* | 23 | 27.71% | 4 | 11.11% | 38 | 23.31% |
| *Acinetobacter baumannii* | 26 | 31.33% | 21 | 58.33% | 9 | 5.52% |
| *Escherichia coli* | 5 | 6.02% | 1 | 2.78% | 43 | 26.38% |
| *Enterococcus faecium* | - | - | - | - | 42 | 25.77% |
| *Staphylococcus aureus* | 8 | 9.64% | 6 | 16.66% | 4 | 2.45% |
| *Pseudomonas aeruginosa* | 4 | 4.82% | 1 | 2.78% | 7 | 4.29% |
| *Enterobacter hormaechei* | 5 | 6.02% | 1 | 2.78% | 4 | 2.45% |
| *Stenotrophomonas maltophilia* | 6 | 7.23% | 1 | 2.78% | - | - |
| *Proteus mirabilis* | 1 | 1.2% | - | - | 4 | 2.45% |
| *Enterococcus faecalis* | - | - | - | - | 5 | 3.06% |
| *Klebsiella oxytoca* | - | - | - | - | 2 | 1.27% |
| *Serratia marcescens* | 1 | 1.2% | 1 | 2.78% | - | - |
| *Morganella morganii* | 1 | 1.2% | - | - | 1 | 0.61% |
| *Providencia stuartii* | 1 | 1.2% | - | - | 1 | 0.61% |
| *Proteus vulgaris* | - | - | - | - | 1 | 0.61% |
| *Citrobacter braakii* | - | - | - | - | 1 | 0.61% |
| *Citrobacter freundii* | - | - | - | - | 1 | 0.61% |
| *Enterobacter asburiae* | 1 | 1.2% | - | - | - | - |
| *Acinetobacter schindleri* | 1 | 1.2% | - | - | - | - |
| Total | 83 | 100% | 36 | 100% | 163 | 100% |

**Table S2.** Distribution of bacteria involved in colonization at 7 days after ICU admission (152 strains)

| | **Pharyngeal exudate** | | **Nasal exudate** | | **Rectal swab** | |
|---|---|---|---|---|---|---|
| | Number | Percentage | Number | Percentage | Number | Percentage |
| *Acinetobacter baumannii* | 26 | 42.62% | 15 | 57.69% | 14 | 21.54% |
| *Klebsiella pneumoniae* | 19 | 31.15% | 10 | 38.46% | 19 | 29.23% |
| *Pseudomonas aeruginosa* | 9 | 14.75% | 1 | 3.85% | 6 | 9.23% |
| *Enterococcus faecium* | - | - | - | - | 14 | 21.54% |
| *Escherichia coli* | - | - | - | - | 6 | 9.23% |
| *Enterobacter hormaechei* | 3 | 4.92% | - | - | 1 | 1.54% |
| *Proteus mirabilis* | - | - | - | - | 3 | 4.61% |
| *Stenotrophomonas maltophilia* | 3 | 4.92% | - | - | - | - |
| *Morganella morganii* | - | - | - | - | 1 | 1.54% |
| *Providencia stuartii* | - | - | - | - | 1 | 1.54% |
| *Staphylococcus aureus* | 1 | 1.64% | - | - | - | - |
| Total | 61 | 100% | 26 | 100% | 65 | 100% |

**Table S3.** Distribution of bacteria involved in colonization at 14 days after ICU admission (59 strains)

| | **Pharyngeal exudate** | | **Nasal exudate** | | **Rectal swab** | |
|---|---|---|---|---|---|---|
| | Number | Percentage | Number | Percentage | Number | Percentage |

| | | | | | | |
|---|---|---|---|---|---|---|
| *Acinetobacter baumannii* | 10 | 40% | 8 | 44.44% | 3 | 18.75% |
| *Klebsiella pneumoniae* | 7 | 28% | 6 | 33.33% | 6 | 37.5% |
| *Pseudomonas aeruginosa* | 5 | 20% | 2 | 11.11% | 2 | 12.5% |
| *Proteus mirabilis* | 1 | 4% | 1 | 5.56% | 2 | 12.5% |
| *Escherichia coli* | - | - | 1 | 5.56% | 1 | 6.25% |
| *Stenotrophomonas maltophilia* | 2 | 8% | - | - | - | - |
| *Enterococcus faecium* | - | - | - | - | 1 | 6.25% |
| *Enterococcus faecalis* | - | - | - | - | 1 | 6.25% |
| Total | 25 | 100% | 18 | 100% | 16 | 100% |

**Table S4.** Distribution of bacteria involved in colonization at 21 days after ICU admission (29 strains)

| | **Pharyngeal exudate** | | **Nasal exudate** | | **Rectal swab** | |
|---|---|---|---|---|---|---|
| | Number | Percentage | Number | Percentage | Number | Percentage |
| *Pseudomonas aeruginosa* | 5 | 35.72% | 3 | 42.86% | 1 | 12.5% |
| *Acinetobacter baumannii* | 3 | 21.43% | 3 | 42.86% | 1 | 12.5% |
| *Klebsiella pneumoniae* | 4 | 28.57% | - | - | 3 | 37.5% |
| *Proteus mirabilis* | 1 | 7.14% | 1 | 14.28% | 1 | 12.5% |
| *Escherichia coli* | 1 | 7.14% | - | - | 1 | 12.5% |
| *Enterococcus faecium* | - | - | - | - | 1 | 12.5% |
| Total | 14 | 100% | 7 | 100% | 8 | 100% |

**Table S5.** Distribution of bacteria involved in colonization at 28 days after ICU admission (18 strains)

| | **Pharyngeal exudate** | | **Nasal exudate** | | **Rectal swab** | |
|---|---|---|---|---|---|---|
| | Number | Percentage | Number | Percentage | Number | Percentage |
| *Pseudomonas aeruginosa* | 3 | 42.86% | 2 | 66.66% | 2 | 25% |
| *Acinetobacter baumannii* | 3 | 42.86% | 1 | 33.33% | 1 | 12.5% |
| *Klebsiella pneumoniae* | 1 | 14.28% | - | - | 3 | 37.5% |
| *Escherichia coli* | - | - | - | - | 1 | 12.5% |
| *Proteus mirabilis* | - | - | - | - | 1 | 12.5% |
| Total | 7 | 100% | 3 | 100% | 8 | 100% |

**Table S6.** Distribution of bacteria involved in colonization at 35 days after ICU admission (8 strains)

| | **Pharyngeal exudate** | | **Nasal exudate** | | **Rectal swab** | |
|---|---|---|---|---|---|---|
| | Number | Percentage | Number | Percentage | Number | Percentage |

| | | | | | | |
|---|---|---|---|---|---|---|
| *Pseudomonas aeruginosa* | 1 | 33.33% | 1 | 33.33% | 1 | 50% |
| *Acinetobacter baumannii* | 1 | 33.33% | 1 | 33.33% | - | - |
| *Klebsiella pneumoniae* | 1 | 33.33% | 1 | 33.33% | 1 | 50% |
| Total | 3 | 100% | 3 | 100% | 2 | 100% |

**Table S7.** Antibiotic resistance patterns of *Enterobacter* spp. involved in colonization (15 strains)

| Antibiotic | Susceptible, standard dosing (S) | | Susceptible, increased exposure (I) | | Resistant (R) | |
|---|---|---|---|---|---|---|
| | n | % | n | % | n | % |
| Piperacillin&Tazobactam | 2 | 13.3% | - | - | 13 | 86.7% |
| Ceftazidime | - | - | - | - | 15 | 100% |
| Ceftriaxone | - | - | - | - | 15 | 100% |
| Cefotaxime | - | - | - | - | 15 | 100% |
| Cefepime | 1 | 6.7% | 1 | 6.7% | 13 | 86.7% |
| Ceftaroline | - | - | - | - | 15 | 100% |
| Ceftazidime&Avibactam | 14 | 93.3% | - | - | 1 | 6.7% |
| Ceftolozane&Tazobactam | 10 | 66.7% | - | - | 5 | 33.3% |
| Ertapenem | 11 | 73.3% | - | - | 4 | 26.7% |
| Imipenem | 11 | 73.3% | - | - | 4 | 26.7% |
| Imipenem&Relebactam | 11 | 73.3% | - | - | 4 | 26.7% |
| Meropenem | 14 | 93.3% | - | - | 1 | 6.7% |
| Aztreonam | 1 | 6.7% | - | - | 14 | 93.3% |
| Ciprofloxacin | 1 | 6.7% | 1 | 6.7% | 13 | 86.7% |
| Levofloxacin | 13 | 86.7% | - | - | 2 | 13.3% |
| Amikacin | 14 | 93.3% | - | - | 1 | 6.7% |
| Gentamicin | 2 | 13.3% | - | - | 13 | 86.7% |
| Tobramycin | 2 | 13.3% | - | - | 13 | 86.7% |
| Trimethoprim&Sulfamethoxazole | - | - | - | - | 15 | 100% |
| Colistin | 15 | 100% | - | - | - | - |

**Table S8.** Antibiotic resistance patterns of *Proteus* spp. involved in colonization (17 strains)

| Antibiotic | Susceptible, standard dosing (S) | | Susceptible, increased exposure (I) | | Resistant (R) | |
|---|---|---|---|---|---|---|
| | n | % | n | % | n | % |
| Ampicillin | - | - | - | - | 17 | 100% |
| Ampicillin&Sulbactam | 2 | 11.8% | - | - | 15 | 88.2% |
| Amoxicillin&Clavulanic acid | 2 | 11.8% | - | - | 15 | 88.2% |
| Piperacillin&Tazobactam | 17 | 100% | - | - | - | - |
| Ceftazidime | 2 | 11.8% | 1 | 5.9% | 14 | 82.3% |
| Ceftriaxone | - | - | - | - | 17 | 100% |
| Cefuroxime | - | - | - | - | 17 | 100% |

| | | | | | | |
|---|---|---|---|---|---|---|
| Cefotaxime | - | - | - | - | 17 | 100% |
| Cefepime | 13 | 76.4% | 2 | 11.8% | 2 | 11.8% |
| Ceftaroline | - | - | - | - | 17 | 100% |
| Ceftazidime&Avibactam | 17 | 100% | - | - | - | - |
| Ceftolozane&Tazobactam | 14 | 82.4% | - | - | 3 | 17.6% |
| Ertapenem | 5 | 29.4% | - | - | 12 | 70.6% |
| Imipenem | - | - | 6 | 35.3% | 11 | 64.7% |
| Meropenem | 16 | 94.1% | - | - | 1 | 5.9% |
| Aztreonam | 16 | 94.1% | - | - | 1 | 5.9% |
| Ciprofloxacin | - | - | - | - | 17 | 100% |
| Levofloxacin | 1 | | - | - | 16 | 94.1% |
| Amikacin | 16 | 94.1% | - | - | 1 | 5.9% |
| Gentamicin | 6 | 35.3% | - | - | 11 | 64.7% |
| Tobramycin | 1 | | - | - | 16 | 94.1% |
| Trimethoprim&Sulfamethoxazole | - | - | - | - | 17 | 100% |
